# Transcriptome-targeted analysis of human peripheral blood-derived macrophages when cultured on biomaterial meshes

**DOI:** 10.1088/1748-605X/abdbdb

**Published:** 2021-02-18

**Authors:** Camilo Mora-Navarro, Emily W Ozpinar, Daphne Sze, David P Martin, Donald O Freytes

**Affiliations:** 1 The Joint Department of Biomedical Engineering, North Carolina State University and University of North Carolina-Chapel Hill, 4208D Engineering Building III, Raleigh, NC, United States of America; 2 The Comparative Medicine Institute, North Carolina State University, Raleigh, NC, United States of America; 3 Tepha, Inc, Lexington, MA, United States of America

**Keywords:** surgical meshes, macrophage polarization, biomaterials, targeted transcriptome, poly-4-hydroxybutyrate (P4HB), polypropylene

## Abstract

Surgical meshes are commonly used to repair defects and support soft tissues. Macrophages (M*ϕ*s) are critical cells in the wound healing process and are involved in the host response upon foreign biomaterials. There are various commercially available permanent and absorbable meshes used by surgeons for surgical interventions. Polypropylene (PP) meshes represent a permanent biomaterial that can elicit both inflammatory and anti-inflammatory responses. In contrast, poly-4-hydroxybutyrate (P4HB) based meshes are absorbable and linked to positive clinical outcomes but have a poorly characterized immune response. This study evaluated the *in vitro* targeted transcriptomic response of human M*ϕ*s seeded for 48 h on PP and P4HB surgical meshes. The *in vitro* measured response from human M*ϕ*s cultured on P4HB exhibited inflammatory and anti-inflammatory gene expression profiles typically associated with wound healing, which aligns with *in vivo* animal studies from literature. The work herein provides *in vitro* evidence for the early transcriptomic targeted signature of human M*ϕ*s upon two commonly used surgical meshes. The findings suggest a transition from an inflammatory to a non-inflammatory phenotype by P4HB as well as an upregulation of genes annotated under the pathogen response pathway.

## 1. Introduction

Suture or mesh biomaterials are used to augment surgical repairs and usually should remain relatively inert, causing minimal inflammation and allowing tissue healing while performing their stabilizing function [1]. Multiple studies have shown that permanent biomaterials such as polypropylene (PP) meshes, while effective for tissue support, can have long-term complications often attributed to inflammation due to a chronic foreign body response [2–4]. Absorbable polymeric scaffolds are alternative types of biomaterials that can provide the same function, but may reduce these complications by slowly degrading, allowing the host tissue to remodel and generating new tissue *in situ*.

Absorbable polymeric scaffolds aim to reduce the magnitude of foreign body response and the potential for long-term complications such as pain, contraction, extrusion, and infection [1]. Several recent clinical studies have documented the success of absorbable scaffolds made from poly-4-hydroxybutyrate (P4HB) as an alternative to PP meshes or animal-derived scaffolds to successfully treat ventral hernias, incisional hernia, reduce donor site bulge, prevent ptosis, and as an alternative to acellular dermal matrices in breast reconstruction [5–7]. A better understanding of how absorbable biomaterials interact with the host response is critical in order to improve patient outcomes and develop improved solutions for wound healing and soft tissue support.

Macrophages (M*ϕ*s) play a critical role in the host response and are among the earliest immune cells to respond to tissue damage and surgical repair sites. In combination with tissue-resident M*ϕ*s, newly recruited M*ϕ*s secrete signals to attract appropriate cell types and orchestrate inflammatory and healing events [8]. During the initial inflammatory phase, neutrophils and M*ϕ*s begin the healing process by removing damaged tissue [9], destroying microorganisms, phagocytizing foreign material and debris, and recruiting inflammatory cells and other cell types needed for repair, angiogenesis, and remodeling [10]. M*ϕ*s are crucial contributors to each of these phases of healing and play important roles in orchestrating the body’s response to an injury or surgical insult [11, 12].

This initial inflammatory response can last several days, followed by a M*ϕ*-centered response to stabilize the tissue and guide the healing process [13, 14]. During the healing phase, local cells begin to produce the needed extracellular matrix and, over a period of weeks to months, the matrix matures and remodels as tissue repair occurs. If initial inflammation caused by the insult or the biomaterial does not resolve, a chronic inflammatory response or other adverse outcomes may result [15]. A biomaterial can provide the necessary mechanical support for surgical intervention without long-term, chronic inflammation, while an absorbable biomaterial can slowly degrade allowing healing and site-specific tissue production by promoting the optimal transition of M*ϕ*s.

M*ϕ*s can be induced *in vitro* into distinct, well-characterized phenotypes ranging from the classically-activated, pro-inflammatory M1-like phenotype to the alternatively-activated, anti-inflammatory M2-like phenotype [16]. In an attempt to classify the M*ϕ*s present during different stages of repair, M2-like M*ϕ*s have been further classified into subtypes such as pro-healing M2a, immunoregulatory M2b, immunosuppressive M2c, and tumor-associated M2d [17, 18]. However, M*ϕ*s in the body exist on a spectrum of phenotypic traits due to the dynamic concentration of cytokines present across the host tissues [19]. *In vitro*, the different phenotypes can be induced or approximated experimentally with specific stimuli, thus providing a convenient experimental cell model that can help study the role of M*ϕ*s in homeostasis, inflammation, remodeling, and healing [20], or in the case of this study, their response to different biomaterials. While gene expression assays have been used to characterize the different M*ϕ*s phenotypes, few studies have been performed to elucidate the targeted transcriptome gene expression of human M*ϕ*s in response to *in vitro* culture on different biomaterial meshes and how this might translate *in vivo.*

We hypothesized that an absorbable P4HB mesh will modulate a particular targeted-transcriptome gene expression in M*ϕ*s isolated from human peripheral blood mononuclear cells (PBMCs) that differs from the response to a permanent PP scaffold. In this work, we investigated the response of non-polarized M*ϕ*s (which we identify as ‘M0s’) and polarized M*ϕ*s (‘M1’, ‘M2a’, and ‘M2c’ *like*-phenotypes) to absorbable and permanent meshes typically used in hernia repair and plastic and reconstructive surgical applications. Our goal was to determine how the materials affect the M*ϕ*’s gene expression by contrasting the transcriptomic targeted cell response when seeded on different biomaterials and compared to four *in vitro* control phenotypes (M0, M1, M2a, and M2c).

In this study, we used a commercially available, 740 gene panel targeted towards innate immunity to compare gene expression profiles for non-polarized and polarized M*ϕ*s. We identified genes with significant changes in their expression in M*ϕ*s cultured on commonly used meshes. The changes in the transcriptome response for M*ϕ*s seeded on P4HB mesh provide clues into a potential transition from an inflammatory gene expression profile to a more anti-inflammatory profile. Since the biomaterial-cell interaction with the host immune system is critical for wound healing applications, these results offer insight into the observed *in vivo* responses to these materials and how we can use such information to guide their clinical use.

## 2. Materials and methods

### 2.1. Materials and reagents

Human buffy coats from five unidentified donors were purchased at different times from the New York Blood Center (New York, NY). The EasySep^™^ Human CD14 Positive Selection Kit was purchased from Stem Cell Technologies (Vancouver, Canada). Heat-inactivated human serum (Human Serum-H) derived from male AB plasma (Cat. #H3667) was purchased from MilliporeSigma (Burlington, MA). Corning^®^ Ultra-Low Binding plastic (UBP), well plates and cell culture flasks, 4-hydroxybutyric acid (4HB) as the sodium salt, and Lipopolysaccharide (LPS) were purchased from Sigma-Aldrich (St. Louis, MO). Cytokines used for the differentiation and polarization of M*ϕ*s, Monocyte Colony-Stimulating Factor (M-CSF), Interferon-gamma (IFN*γ*), Interleukins 4, 10, and 13 (IL4, IL10, and IL13, respectively), were obtained from Peprotech (Rocky Hill, NJ).

GalaFLEX^®^ P4HB mesh and fatty acid (FTY) residues extracted from the P4HB were obtained from Tepha, Inc. (Lexington, MA). Prolene^®^ PP-mesh, Vicryl^®^, and Mersilene^®^ were manufactured by Ethicon (Somerville, NJ). TIGR^®^ mesh was manufactured by Novus Scientific (Uppsala, Sweden). SERI^®^ was manufactured by Sofregen Medical (Medford, MA) and Alloderm^®^ was manufacture by Allergan (Dublin, Ireland).

The use of the NanoString Technologies Prep Station and Digital Analyzer instruments was provided by the Lineberger Comprehensive Cancer Center (LCCC) at the University of North Carolina-Chapel Hill School of Medicine.

### 2.2. Scanning electron microscopy (SEM)

Samples from P4HB and PP meshes were sputter coated with a Denton Vacuum Desk V sputter coater using a gold/palladium target for a total of 90 s (3 × 30 s intervals to avoid raising the temperature of the samples) and on 45 kVp intensity. Samples were imaged with a Hitachi S-3400 N-II SEM with Secondary Electron Detector, Accelerating Voltage 10 kV, under high vacuum <1 Pa.

### 2.3. Isolation of monocytes and M*ϕ*s differentiation

CD14^+^ monocytes were isolated from human PBMCs and differentiated into M*ϕ*s as previously described [21, 22].

Heparinized blood was diluted with 1 mM EDTA in 1× Dulbecco’s PBS (DPBS), carefully overlaid onto Lymphocyte H Cell Separation Media (Cedarlane, Burlington, NC) and spun down at 400 g-forces for 20 min. The PBMC layer was removed with a transfer pipette, washed three times with EDTA/DPBS and centrifuged for 150 g-forces for 10 min each. Using the Human CD14 Positive Selection Kit, CD14^+^ cells were isolated from the PBMCs according to the manufacturer’s instructions. Briefly, the cell density was adjusted at 1.0 × 10^8^ cells ml^−1^ and EasySep^™^ positive selection cocktail was added at 100 μl ml^−1^ for 15 min. EasySep^™^ magnetic particles were then added at 50 μl ml^−1^ and incubated for 10 min. Using ‘The Big Easy’ EasySep^™^ Magnet (Stem Cell Technologies), the CD14^+^ cells were isolated by incubating the cell suspension three times for 5 min each and gently removing the supernatant.

After isolation, 0.5–1.0 × 10^6^ cells per antibody were fixed in FACS buffer (0.5% bovine serum albumin (BSA) in 1 mM EDTA in 1× PBS) for 15 min. The cells were then stained (1:100) for 15 min at 4 °C with either a mouse anti-human CD14:APC antibody (BioRad, Hercules, CA, Cat #MCA596APCT), CD34 mouse anti-human:PE (Fisher Scientific, Cat. #BDB560941), or CD68 mouse anti-human:FITC (Fisher Scientific, Cat. #BDB562117). Isotype controls included anti-IgG1 *κ* Mouse:PE (Fisher Scientific, Cat. #BDB555749), anti-IgG2b, *κ* Mouse:FITC (Fisher Scientific, Cat. #BDB555057) and mouse IgG2a:APC (BioRad, Cat #MCA929APC) antibodies. Samples were then washed and fixed with 4% formaldehyde for 15 min, washed and suspended in FACS buffer. Flow cytometry was performed on a BD^™^ LSR II (Becton, Dickinson and Company, Franklin Lakes, NJ) and analyzed using BD FACSDiva^™^ software, see supplemental figure s1 (available online at stacks.iop.org/BMM/16/025006/mmedia).

The isolated CD14^+^ cells were cultured at 1.0 × 10^6^ cells ml^−1^ in low attachment flasks (Corning, Corning, NY) **[**23, 24]. Media used to culture the monocytes and M*ϕ*s was composed of RPMI Media 1640 with GlutaMAX^™^ supplemented with 10% Human Serum-HI and 1% Penicillin/Streptomycin (Thermo Fisher Scientific, Waltham, MA) (‘human M*ϕ* media’). M-CSF was added at 20 ng ml^−1^ to the media to push the CD14+ cells into non-polarized M*ϕ*s (‘M0’) for 5 d with a media change on Day three of culture.

### 2.4. M*ϕ* polarization

On Day five of culture, M*ϕ*s were seeded at 1.0 × 10^6^ cells ml^−1^ with 20 ng ml^−1^ M-CSF into UBP six-well plates. M*ϕ*s that did not receive any other cytokines (non-polarized), only fresh media change, are continually referred to as M0. M*ϕ*s that were polarized with 100 ng ml^−1^ of LPS and 100 ng ml^−1^ of IFN*γ* are referred to as ‘M1’. M*ϕ*s that were polarized with 40 ng ml^−1^ of IL4 and 20 ng ml^−1^ of IL13 were designated ‘M2a’. M*ϕ*s polarized with 40 ng ml^−1^ of IL10 were referred to as ‘M2c’.

### 2.5. Seeding of the biomaterials

#### 2.5.1. Biomaterial screening attachment

7 mm diameter disks of each biomaterial were made with a sterile biopsy punch (Acuderm, Fort Lauderdale, FL) and attached to 96 well plates (Corning). For the material screening experiments, biomaterials were attached using a 15 μl fibrin clot comprised of 2% fibrinogen in 1 × DPBS and thrombin (Sigma-Aldrich, St. Louis, MO) at 100 U ml^−1^ at a 5:1 ratio as reported by [25]. A fibrin clot alone was used as a control. The attached scaffolds were stored in 100 μl of 1 × DPBS at room temperature until ready for cell seeding.

#### 2.5.2. PP and P4HB mesh preparation test

The fibrin clot was not used in any of the subsequent tests for the direct comparisons of PP (PROLENE Mesh, Ref PMII, 3 × 6”, Lot LAH068, Exp. 2021–12-31), P4HB (GalaFLEX Scaffold, Ref GP0408, 10 × 20 cm, Lot 200 354, Exp. 2023–05-31), and UBP. Rather, two layers of 12 mm diameter disks of the meshes (to provide a higher surface area available for cell attachment) were arranged and placed in a 48 well plate. The materials were stored in 400 μl of 1 × DPBS at room temperature until cells were ready for cell seeding.

#### 2.5.3. Mϕ seeding and polarization

M*ϕ* were detached from the UBP flasks with Accutase (Thermo Fisher Scientific) for 5 min at 37 °C on Day 5 of culture, collected, and counted using Countess^™^ II Automated Cell Counter (Thermo Fisher Scientific). Cells were suspended at a cell density of 1.0 × 10^6^ cells ml^−1^, the DPBS was removed from the scaffolds, and 2.0 × 10^5^ cells were seeded onto each scaffold in 200 μl of human M*ϕ* media to each well with 20 ng μl^−1^ of M-CSF. Non-polarized M0s (M*ϕ* + M-CSF) or M*ϕ*s exposed to the cytokines required for the polarization controls (i.e. M1-, M2a-, and M2c-like phenotypes) were also seeded onto UBP alone.

The plates were kept at 37 °C with 5% CO_2_ concentration for 48 h until Day 7 of culture. The sample collection was done on Day 7 of culture by carefully removing the scaffolds from the media container and immersed twice into DPBS to wash out non-attached M*ϕ*s. Cells attached to P4HB and PP were stained using CellMask^™^ Deep Red Plasma Membrane Stain at 5 μg ml^−1^ for 30 min following manufacturer protocol. The samples were gently washed in DPBS 1× twice before imaging using fluorescence microscopy with a Revolve microscope (Echo, San Diego, CA). Separate scaffolds with adherent cells were used for RNA isolation.

#### 2.5.4. Mϕ seeding on UBP upon 4HB and FTY exposure

Since P4HB is absorbable and may hydrolyze to release low molecular weight species, M0s were also seeded onto UBP along with the FTY monomer 4HB and extractable FTY residues from the P4HB polymer at the following concentrations 4HB (5 mM) and FTY (5 μg ml^−1^). Samples were analysed on Day 7 n using 350 μl of TRK Lysis Buffer provided by the E.Z.N.A. Total RNA Kit I (Omega Bio-tek, Norcross, GA) and stored at −80 °C.

### 2.6. RNA isolation and nanostring gene expression

After a gentle immersion in DPBS to remove non-attached cells as described in section 2.4.3, the scaffolds with adherent cells were placed into microcentrifuge tubes containing 350 μl TRK lysis buffer and stored at −80 °C for no longer than a week. RNA was isolated via the E.Z.N.A. Total RNA Kit I according to the manufacturer’s instructions. The quality of the RNA recovered was assessed by using Agilent Tapestation 2200TM Quality Assessment. Gene expression was measured using nCounter^®^ Myeloid Innate Immunity Gene Expression Panel V2.0 (Seattle, WA), using at least 100 ng of RNA per sample. Briefly, the RNA was hybridized overnight at 65 °C with reporter and capture probes, including probes for several positive controls and housekeeping genes. The RNA and probes were then washed and attached to a nCounter cartridge in the nCounter^®^-NanoString Technologies Prep Station. The cartridge was then read in the nCounter Digital Analyzer.

### 2.7. Data analysis

NanoString data was imported into the nSolver^™^ software V 4.0 (NanoString Technologies, Seattle WA) and analyzed using the nCounter Advanced Analysis plugin. Normalization, differential expression, and gene set analysis (GSA) were conducted on the raw data using nSolver [26]. Briefly, the software normalizes the data to the geometric mean of housekeeping genes selected using the geNorm algorithm [27]. The data was sorted by using housekeeping genes with counts larger than 100. The differential expression calculated the Log2 fold change (FC) values of the normalized data and the p-values adjusted using the Benjamini-Yekutiel method, which estimates the false discovery rate assuming there may be some biological connection between genes. GSA computed the directed global significance scores relative to a selected control. This value represented the overall differential expression of a particular gene set calculated using the signed square root of the mean squared t-statistic of genes. Full details of these algorithms can be found in the nCounter Advanced Analysis 2.0 Plugin for nSolver Software User Manual. The principal component analysis (PCA) was conducted with a custom R script (R version 3.6.1, R Studio version 1.2.5001) using the ‘prcomp’ function and normalized nSolver data [28]. Graphs were prepared using the ‘ggplot2’ and ‘Complex Heatmap’ packages in R [29, 30].

## 3. Results

### 3.1. M0s (M*ϕ* + M-CSF) response to biomaterials

**Figure 1.**
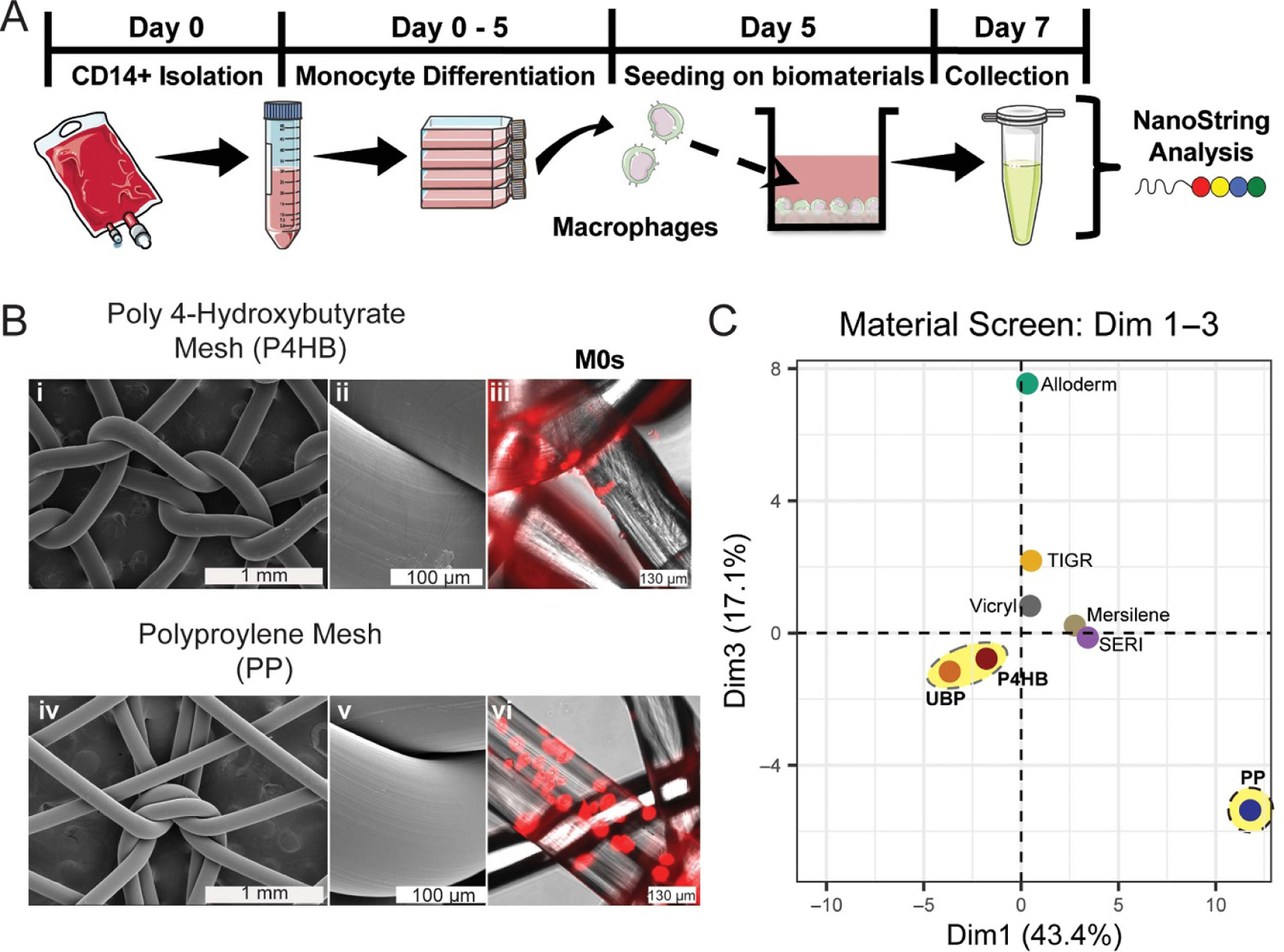
The experimental design approach and material screening. (A) Timeline for cell isolation, culture, seeding, and transcriptome analysis. (B) Images representing the microview of P4HB (absorbable) and PP (permanent) meshes. (i),(ii),(iv), and (v) SEM Images. (iii) and (vi) Plasma membrane fluorescently stained images of M0-like phenotype cultured on P4HB and PP. Plasma membranes were stained with cellMask^™^ deep red. (C) PCA of M0s (M*ϕ* + M-CSF) cultured on various biomaterials. Each point represents the mean coordinates of the individual samples for each biomaterial. Each sample is the biological average from one reading of three replicates pooled together (*n* = 3).

The targeted transcriptional response of M*ϕ*s to several different biomaterial scaffolds (i.e. UBP, GalaFLEX^®^ (P4HB), Prolene^®^ (PP), TIGR^®^, Vicryl^®^, Mersilene^®^, SERI^®^, and Alloderm^®^), was determined using the experimental design shown in figure 1(A). Human CD14+ were isolated from PBMCs and then cultured in media supplemented with M-CSF to differentiate them towards M*ϕ*, see supplemental figure s1 for CD14+ FACS identification. After 5 d of incubation time, M0s (M*ϕ*s + M-CSF) were seeded onto the biomaterials for 48 h (endpoint of cell culture 7 d). The RNA was then isolated and processed via NanoString sequencing with a panel for innate immunity to evaluate the targeted transcriptional response.

Figure 1(B) shows representative SEM images for P4HB (absorbable) and PP (permanent) meshes. Notably, the knitted meshes show smooth monofilament fibers with a diameter around 150–175 μm. SEM images show a mainly uniform filament with similar substrate topography, curvature and contact areas. The M*ϕ*s cultured on these materials were not subjected to any external loads or mechanical stimuli.

The other biomaterials tested, table 1, include biomaterials of different compositions (TIGR, SERI, Mersilene, and Vicryl) and decellularized human tissue (Alloderm). PCA using the population Z score of the normalized transcriptome data (figure 1(C)), was conducted to evaluate the M0s transcriptome response of the biomaterials relative to UBP control. Overall, most mesh materials localized to the middle of the dimension (Dim) 1—Dim 3 coordinate plane, which describes 60.5% of the transcriptome data variance (supplemental figure s3, other dimensions from the PCA).

Alloderm, a human-derived acellular dermal matrix, had a differential response corresponding to a large increase in the Dim 3 axis with little change in Dim 1. Two absorbable meshes, TIGR (made from two copolymers based on glycolide, lactide, and trimethylene carbonate) and Vicryl (polyglactin 910), also saw an increase in Dim 3 with little change in Dim 1, but to a much lower extent.

On the other hand, transcriptome data for M0s cultured on SERI (absorbable silk-based mesh) and Mersilene (permanent polyester mesh) were located in a common region with an observable change in Dim 1. M0s cultured on UBP localized to quadrant III with only absorbable P4HB located closely in the same quadrant. PP mesh, a permanent biomaterial with a similar monofilament fiber structure (figure 1(B)) to P4HB mesh, in the PCA plot separated to the bottom right of the IV quadrant. This PCA suggests that P4HB and UBP are most similar on these axes, and a large differential transcriptome response was seen in M0s cultured on the absorbable P4HB compared to the M0s on PP.

### 3.2. M0 response to P4HB mesh

**Figure 2.**
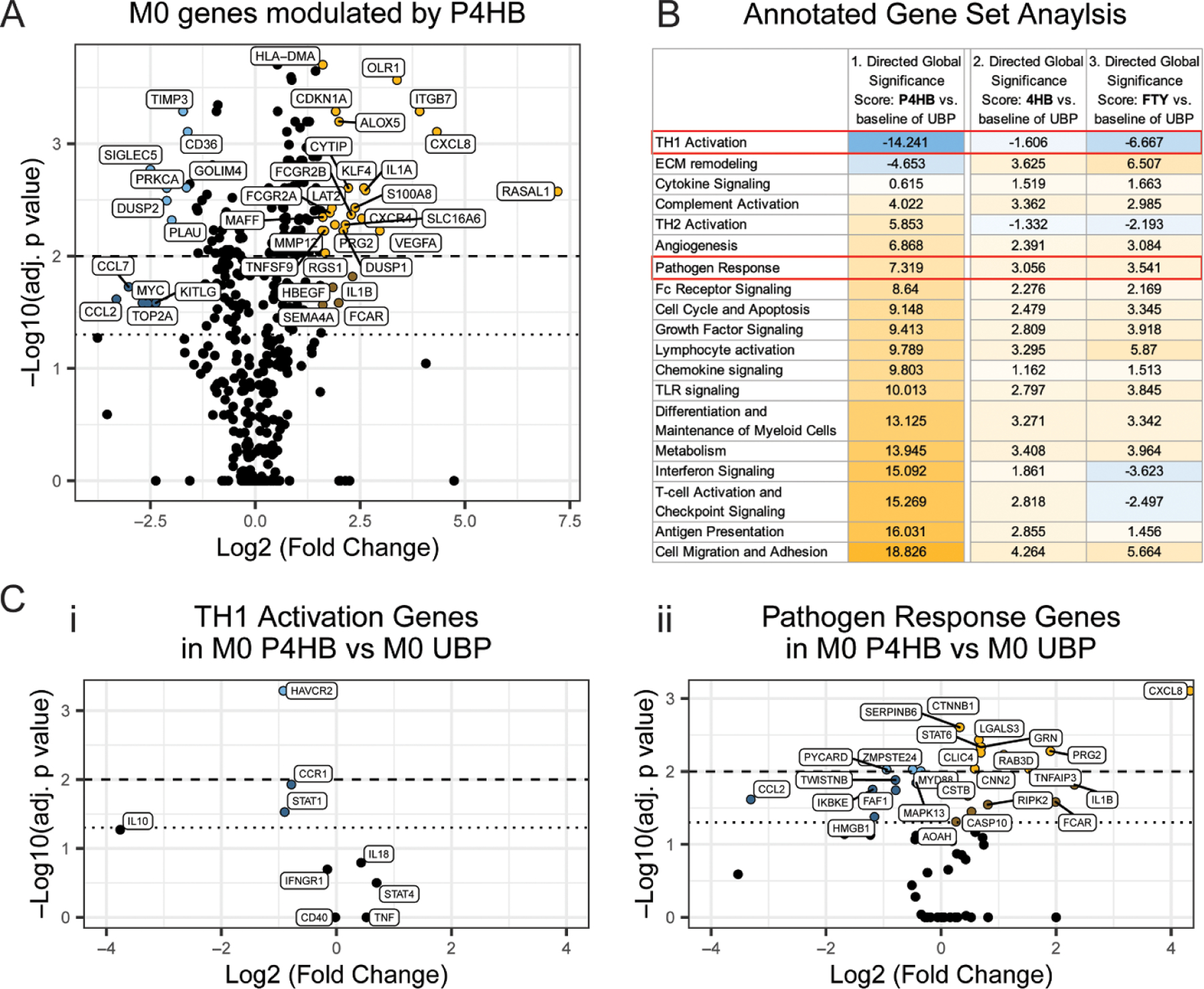
M0 (M*ϕ* + M-CSF) response to P4HB mesh. (A) Volcano plot representing the -Log 10 adjusted p-value and Log2 FC gene expression for M*ϕ*s cultured on P4HB vs UBP. (B) GSA based on the differential expression testing of P4HB (column 1), the 4HB monomer (column 2), and fatty acid residues extracted from the P4HB (FTY, column 3). (C) Volcanos plots referencing the annotated pathways (i) TH1 Activation and (ii) Pathogen Response from the GSA table. The data represents n = 3 independent biological replicates. ‘- - -’ and ‘…’ represent the alpha for -Log10 adj. *p*-values equal to 0.01 and 0.05 respectively.

A more in-depth evaluation of the targeted transcriptome for M0s cultured on the absorbable P4HB mesh referenced to M0s cultured on UBP via NanoString mRNA expression profile is depicted in the volcano plot in figure 2(A). Here, a group of 133 genes showed a shift to the right demonstrating upregulation by the P4HB mesh compared to UBP (adj. *p*-value < 0.05 and Log2 (P4HB vs UBP) > 0). From this significantly upregulated group, 27 genes (represented in yellow) have a FC larger than three. *CXCL8* and *RASAL1* were two genes with the highest statistically significant FC observed. Meanwhile, from the 77 genes significantly downregulated by the material, 12 genes (represented in blue) presented a FC reduction equal or lower than a third compared to the UBP condition. To better understand the effect of this differential expression, a GSA was conducted to determine how P4HB-mesh affected each annotated pathway. This analysis is shown in figure 2(B), where the directed global significance scores are listed with a color scale to highlight the up or downregulation (yellow or blue color, respectively) of the annotated pathway (see supporting file LBL-10 397-01_nCounter_Hs_Myeloid_Innate_I, used with permission from NanoString Technologies, Inc).

Overall, P4HB led to major changes in the differential expression, with all but two pathways upregulated. TH1 activation annotated pathway was the most significantly downregulated pathway. Along with this pathway, the extracellular matrix (ECM) remodeling pathway was also downregulated. A volcano plot for the genes annotated in the ECM remodeling pathway can be found in the supplemental figure s4(A).

On the other hand, genes within the cell migration and adhesion pathway were upregulated in P4HB compared to the UBP reference (figure 2(B) column 1, Volcano plot for Cell migration and adhesion pathway in supplemental figure s5). Similarly, the pathogen response pathway showed an upregulation of the genes clustered in this pathway. The pathogen response is of particular interest as recent reports show P4HB meshes elicit a pro-healing immune response and increased expression of antimicrobial peptides in animal models and with isolated macrophages [34]. Thus, the TH1 activation and pathogen response annotated pathways, enclosed in red, were selected for further analysis.

Next, the low molecular weight species 4HB and extracted FTY were tested with M0s to determine if the observed response resulted from the degradation product of the polymer P4HB or FTY residues present in the P4HB polymer from its production. GSA, seen in figure 2(B), columns 2 & 3, revealed similar trends with TH1 activation associated genes generally downregulated, and genes associated with pathogen response generally upregulated. However, there were small changes in the overall gene expression between 4HB and FTY under the conditions tested when compared to the full effect of the P4HB mesh.

The volcano plot in figure 2(C(i)) shows the genes associated with the TH1 activation annotated pathway for M0 cultured on P4HB mesh. The three most significantly downregulated genes (*HAVCR*, *STAT1*, and *CCR1*) had a Log2 FC of approximately −1.9 to −1.7. It is noticeable that *IL10* was downregulated, however, it was not considered significant with an adjusted p-value of 0.0535. Figure 2(C(ii)) presents the Pathogen response pathway with the significant upregulation of 15 genes, including *CXCL8*, *STAT6*, and *IL1B* contrasted to 9 significantly downregulated genes such as *CCL2*, *PYCARD*, and *ZMPSTE24*.

### 3.3. M*ϕ*s polarization on P4HB mesh upon polarizing stimuli

The effect of P4HB on M*ϕ* polarization was evaluated by performing an analogous GSA with polarized M1, M2a, and M2c like-phenotypes in reference to the M0s cultured on UBP.

**Figure 3.**
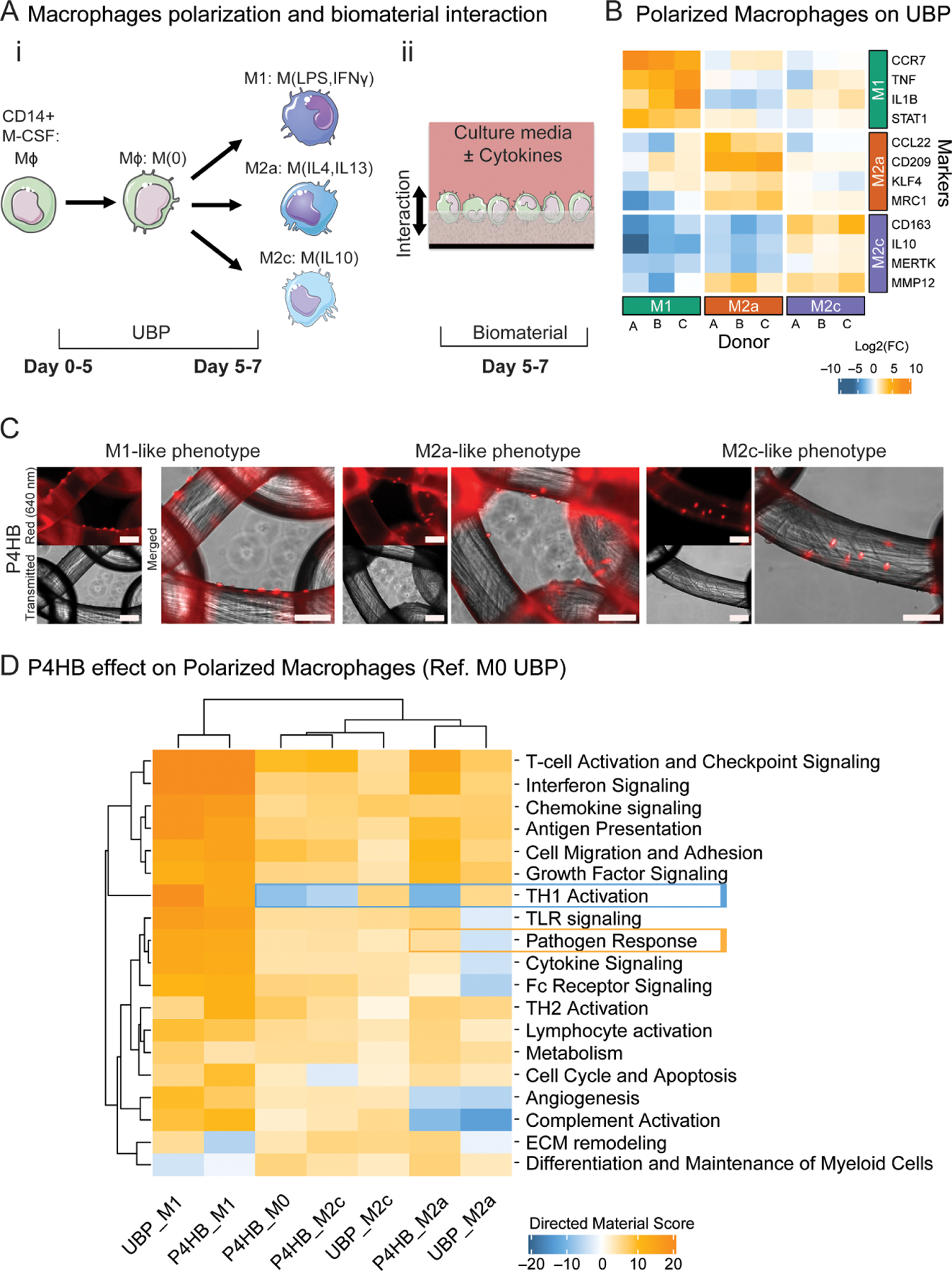
M0 (M*ϕ* + M-CSF), M1 (LPS, IFN*γ*), M2a (IL4, IL13), and M2c (IL10) M*ϕ* response to P4HB (A) Schematic for (i) M*ϕ* polarization (ii) interaction between M*ϕ* and biomaterial (B) Polarization markers for M1, M2a and M2c-like phenotypes in M*ϕ* cultured on UBP across three donors. Colors represent the Log2 FC value referenced to non-polarized M0 UBP control corresponding to the same donor and gene. (C) Fluorescent images of M1, M2a and M2c-like phenotype M*ϕ*s cultured on P4HB. For each condition the top left image is stained for the plasma membrane (Deep Red), the bottom left is transmitted light, and the right is a merge of the two. Scale bar = 130 μm. (D) Directed global significance scores for polarized M*ϕ*s. Scores calculated for M0, M1, M2a, M2c like-phenotype in M*ϕ*s on P4HB and UBP referencing a baseline of M0 on UBP.

Figures 3(A(i) and (ii)) illustrate cell culture conditions and cytokine supplementations to the culture media to polarize the M*ϕ*s (described in section 2.5.3 M*ϕ* Seeding and Polarization), which were then transferred to the biomaterial or UBP as control. M*ϕ*s polarized on UBP used during this work were compared across three different donors (A, B, and C) and the expression of representative gene markers from each like-phenotypes are shown in figure 3(B). As expected, all representative gene markers had higher expression in their respective M*ϕ* like-phenotypes across all three donors shown in figure 3(B) as the diagonal yellow-orange color pattern. Additional genes identified from this analysis that are highly expressed in M*ϕ*s polarized on UBP can be found in supplemental figure s2.

M*ϕ*s, seeded and polarized on P4HB and PP meshes, are shown in figure 3(C) shown in red at Day 7. The images show that polarized M*ϕ*s attach to the biomaterials at the experimental endpoint, which is the same time used for RNA isolation.

Figure 3(D) shows the directed global significance scores of both P4HB and UBP for each polarization normalized to M0s cultured on UBP. Overall, the P4HB absorbable material did not interfere with the polarization behavior of M*ϕ*s cultured except for specific pathways and conditions.

M1 like-phenotype controls (UBP_M1) clustered with P4HB_M1, with most pathways upregulated in both conditions. However, the genes found in the ECM remodeling pathway were downregulated by P4HB (volcano plot is shown in supplemental figure s4(b)). In the case of the M2a like-phenotype, the genes annotated in the TH1 activation pathway in P4HB_M2a were downregulated, contrasting the upregulation pattern typically seen in UBP_M2a. This effect can also be seen in the P4HB_M2c condition whose annotated genes show an overall downregulation compared to the upregulated reference, UBP_M2c. The downregulation of the TH1 activation pathway by P4HB was also seen in M0s, indicating that the change in gene expression relative to UBP is conserved even in the presence of polarizing cytokines. Meanwhile, the pathogen response pathway was upregulated in P4HB_M2a conditions contrary to what was observed for its control UBP_M2a where the genes annotated into the pathway were downregulated. The heat map also shows other annotated pathways such as toll-like receptor (TLR) signaling, cytokine signaling, and Fc receptor signaling that behave in a different manner than the UBP control.

### 3.4. Pathogen-related gene transcriptome between P4HB vs PP

**Figure 4.**
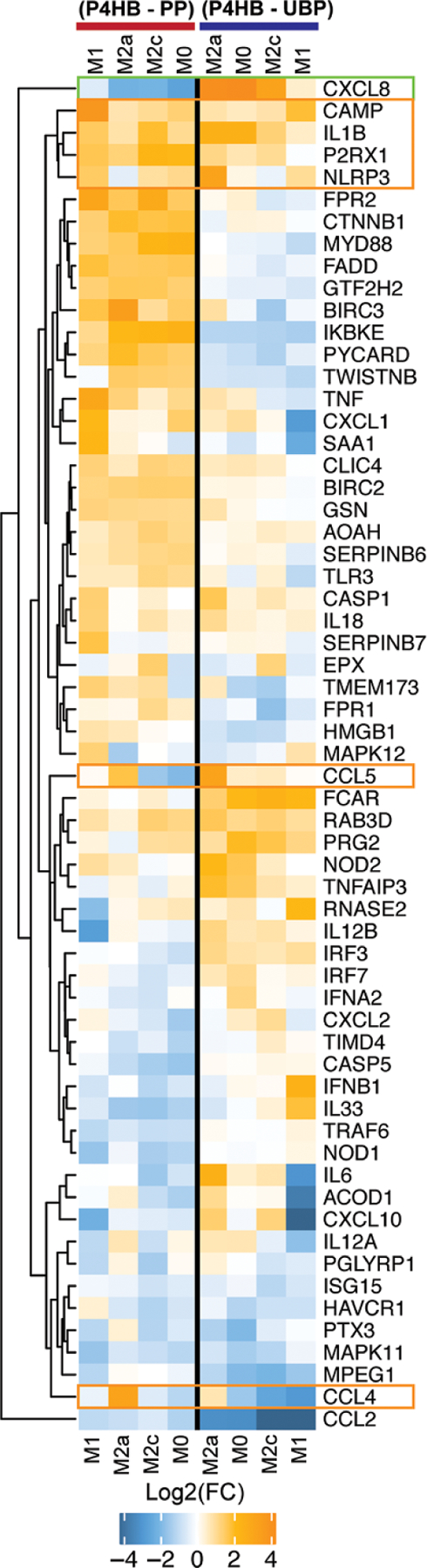
Heatmap for P4HB vs PP and P4HB vs UBP. The heat map shows the pathogen annotated genes. M0s and M*ϕ*s upon polarization signaling are presented in the columns. M0 (M*ϕ* +M-CSF), M1 (LPS, IFNγ), M2a (IL4, IL13), and M2c (IL10).

The Log2 FC for the genes annotated in the pathogen response pathway was extracted and contrasted as follows: P4HB vs PP and P4HB vs UBP using a dendrogram analysis presented in figure 4. *CXCL8* was less expressed in P4HB than in PP, but it was significantly upregulated for M*ϕ*s cultured and polarized on P4HB than on UBP. Interestingly, *CAMP* was upregulated, but these changes were not considered before due to the low statistical significance in the general data analysis. Also, *IL1B*, *P2RX1*, and *NLRP3* were upregulated regarding both P4HB and polarization. In the middle section of the dendrogram, *CCL5* was upregulated for both references under M2a-like conditions. Similarly, located in the bottom section of the dendrogram, *CCL4* shows the same trend of upregulation in P4HB under M2a cytokine polarization conditions.

At the bottom, *CCL2* was downregulated for all the conditions tested. The pathogen response profile under various polarization conditions prompted further global evaluation of PP and P4HB meshes.

### 3.5. Particularities on M*ϕ* response between P4HB and PP

**Figure 5.**
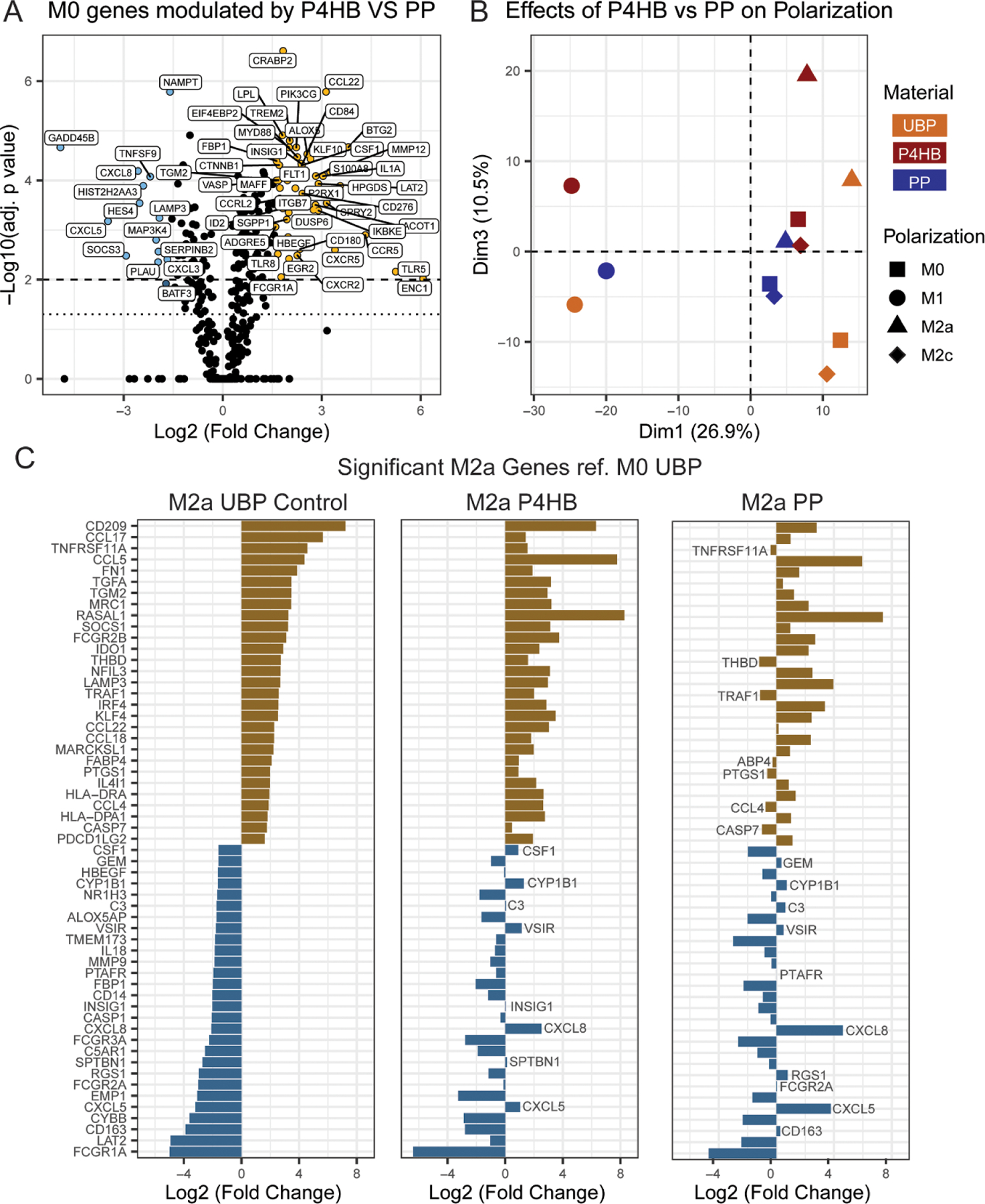
Macrophage (M*ϕ*) response to P4HB mesh compared to PP. (A) Volcano plot representing the Log2 FC gene expression for M0s cultured on P4HB vs PP. The data represents *n* = 3 independent replicates. ‘- - -’ and ‘…’ represent the threshold for −Log10 adj. *p* values equals 0.01 and 0.05 respectively. (B) Biplot with dimensions 1 and 3 of PCA of non-polarized and polarized M*ϕ* (Shape: M0, M1, M2a, and M2c) on various materials (Color: UBP, P4HB, and PP). (C) FC differences of statistically significant genes with an FC greater than 3 in M2a-like M*ϕ*s cultured on UBP referenced to M0s culture on UBP. The expression of M2a-like M*ϕ* cultured on P4HB and PP for the same genes are also presented. The data represents the mean *±* SEM. M0 (M*ϕ* + M-CSF), M1 (LPS, IFNγ), M2a (IL4, IL13), and M2c (IL10).

To compare the M*ϕ* response to absorbable P4HB with a physically comparable permanent mesh, the differential expression of the M0s transcriptome in P4HB vs PP was analyzed (Volcano plot shown in figure 5(A)). 154 significantly upregulated genes were identified (adj. p-value <0.05), with 45 genes having a 3-FC or greater. There were also 77 significantly downregulated genes, with 14 genes having an FC reduced to 1/3x or lower. Similar to the M0 response seen in P4HB vs UBP, a trend towards upregulation was observed when contrasting P4HB vs PP. To assess how these changes may affect polarization, a PCA was performed with M0, M1, M2a, and M2c-like phenotypes cultured on either a UBP control, PP, or P4HB seen in figure 5(B).

Dim 1 of this PCA describes 26.9% of the variance and separates the M1s (circle shapes) to the left, indicating that polarization strongly affects the gene expression of M*ϕ* cultured on all three materials. The M1s cultured on P4HB further separates into the upper half of the plot in Dim 3. The M0s and M2c-like phenotypes (square and diamond shapes respectively) of each material group together, though in distinct locations. This suggests that the polarization to M2c does not have a large effect on gene expression selected in this innate immunity panel compared to the variance caused by each material. Dim 3 separates the polarized phenotypes cultured on P4HB to the top half of the plot (quadrant I), while PP and UBP group in quadrant IV.

M2a-like phenotype (triangle shape) cultured on all three materials is located in the upper half of Dim 3, specifically in quadrant I alongside the M0s and M2cs cultured on P4HB. In P4HB and UBP, polarization towards an M2a-like phenotype results in a large shift up or increase in Dim3 compared to M0s of the corresponding material. However, this increase is not seen when polarizing to M2as on PP. Indeed, PP seems to reduce the change in gene expression associated with Dim 3 when polarizing to M2as, resulting in less separation from the corresponding M0s. Notably, it groups closely with M0s and M2cs cultured on P4HB.

Further analysis into the potential genes that are generating this difference in M2as cultured on UBP, P4HB, and PP was performed and presented in figure 5(C). Genes that were significantly changed (*p*-value < 0.05) with a FC greater than three when polarized to M2a-like phenotype cultured on UBP (M2a UBP ref. M0 UBP) were selected and plotted for all three materials. In M2as cultured on UBP, 29 genes were upregulated vs 28 downregulated (gold and blue color, respectively). Of the 29 upregulated genes, all genes were similarly upregulated in P4HB; however, seven genes were downregulated in PP. Of the 28 downregulated genes in M2as cultured on UBP, 8 genes were upregulated by P4HB and PP upregulated 10 genes. Overall, gene expression in P4HB tracks more closely to UBP than PP.

## 4. Discussion

Surgical meshes are commonly used to support tissue recovery by providing mechanical and structural support for breast reconstruction, hernia repair, reconstruction of the pelvic floor, and other applications [35, 38]. The local tissue response towards surgical meshes (i.e. absorbable or permanent) remains important for proper wound healing or optimal material integration [39–41]. Any biomaterial implanted into a site of injury will interact with the host’s dynamic wound environment where M*ϕ*s are critical cells with roles in inflammation, angiogenesis, extracellular matrix remodeling, and cell recruitment [42]. As M*ϕ*s adhere and interact with the surface of an implanted biomaterial, it is crucial to understand the M*ϕ*’s phenotypical changes when exposed *in vitro* to commercially available meshes [43]. The present study focused on two clinically available biomaterials: one absorbable (P4HB) and the other permanent (PP) (using ultra-low binding tissue culture plastic (UBP) as a control).

In addition to the material’s composition, M*ϕ* polarization can also be affected by material properties such as contact area, stiffness, surface chemistry, and material topography [44–46]. As the M*ϕ*s cultured on the materials were not subjected to macroscopic mechanical stimuli, the mechanical properties of the meshes fell out of the scope of this work but some reports show potential similarities that may be of interest in future studies [47–49].

Initially, to understand how the M*ϕ*s respond to different types of biomaterials, we measured the expression profile of targeted genes by M0s (M*ϕ*s + M-CSF) when cultured *in vitro* on the surface of several clinically available biomaterials (biomaterials summarized in table 1) [31–37]. The screenings presented in figure 1(C) and supplemental figure s3 show a difference between permanent, absorbable, and naturally derived biomaterials. In order to understand the response of M*ϕ*s towards different types of biomaterials, we first focused on biomaterials with varying surfaces such as multifilament biomaterials knitted into meshes (Mersilene^®^, SERI^®^, TIGR^®^ and Vicryl^®^) and monofilament meshes such as P4HB and PP (figure 1(B) and table 1) [50]. Our screening also included representative biomaterials from synthetics (PP, P4HB, Mersilene^®^, TIGR^®^, and VICRYL^®^) and biological sources (Alloderm^®^ and SERI^®^). P4HB is a polyester obtained from a bacterial organism and PP is a polyolefin produced using catalytic reactions [51]. In general, these thermoplastic materials are extruded into fibers and converted into meshes, while silk-derived fibroin fibers are sourced from cocoons of the *Bombyx mori* silkworm, cleaned and converted into meshes. Alloderm^®^ is sourced from cadaveric human skin and was chosen as a representative absorbable acellular matrix [52]. Previous studies have reported, to some extent, the M*ϕ* response towards these biomaterials, but the *in vitro* response of human M*ϕ*s towards P4HB based biomaterials remains mostly under-explored [34, 53–59].

Targeted transcriptome comparison using PCA of non-polarized M*ϕ*s (figure 1(C)) showed that multifilament materials clustered together, Alloderm^®^ and PP clustered away from the rest of the materials, and P4HB clustered with UBP. Interestingly, PP showed a significant phenotype change, separating from both mono- and multi-filament mesh materials. P4HB meshes also have absorbable and degradative properties, while PP is permanent and non-degradable (table 1). While some material properties were controlled, such as shape and topography, the differential M*ϕ* transcriptome changes between P4HB and PP could be attributed to the polymer composition, substrate stiffness, or surface chemistry. A previous study showed how P4HB-based meshes could promote neovascularization and reduce inflammatory and fibrotic responses when implanted in a porcine hernia model [60]. Our *in vitro* characterization results provide further evidence that the absorbable P4HB scaffold could influence the expression of pro- and anti-inflammatory genes in M0s suggesting potential immunomodulatory effects (figure 2(A)). However, this remains to be entirely determined by direct comparison with *in vivo* responses.

The transcriptome-targeted analysis performed suggests that P4HB stimulates the M0s towards a co-expression of genes associated with both pro-inflammatory and anti-inflammatory phenotypes. P4HB upregulated a group of genes associated with inflammatory phenotypes (e.g. *ITGFB7*, *IL1A*) as well as genes related to anti-inflammatory phenotypes such as *MMP12, FCGR2B, and KLF4*. Interestingly, P4HB has been shown to upregulate *KLF4*, which is a critical transcription regulator factor of M*ϕ* polarization, suggesting that P4HB could stimulate a transition to an anti-inflammatory state [61]. This anti-inflammatory effect by P4HB resulted in the downregulation of genes annotated as part of the TH-1 Activation group, where *HAVCR2*, *CCR1*, and *STAT1* were downregulated ~3–4 fold (figure 2(C(i))). Generally, *STAT1* and *STAT6* are thought to be antagonistic, with a downregulation of STAT1 helping to shift the M*ϕ* phenotype from M1-like to M2-like phenotype [62–65]. This gene expression profile is reflected in the transcriptome data with the downregulation of STAT1 and upregulation of STAT6 by P4HB, as shown in figures 2(C(i)) & (C(ii)) for the TH-1 activation and pathogen response genes, respectively.

Clinical studies as well as *in vivo* animal studies have suggested that the use of P4HB may enhance the host defense response against microbial infections [6, 66–68]. In our *in vitro* platform, P4HB mesh affected the expression of genes annotated in the pathogen response pathway for all M*ϕ* polarizations, as seen in figure 3(C), but the specific mechanism has yet to be determined. *STAT6* and *CXCL8* (IL-8), genes annotated in the pathogen response pathway, were significantly upregulated in M0s by P4HB. Furthermore, IL-8 is a chemokine involved in the recruitment of neutrophils and other leukocytes in response to pathogens, which may provide insights on the potential mechanism of the antimicrobial effects shown by P4HB. The data in figure 4 also showed an upregulation of *CAMP* (Cathelicidin also called LL-37), an antimicrobial peptide, whose upregulation in mice (*Cramp gene)* provides resistance to bacterial contamination [58, 66, 69]. *CCL5*, another gene in the pathogen response that acts as a chemokine for lymphocytes recruitment as well as activates antigen-specific T-cells, was upregulated in M2a P4HB compared to PP or UBP, suggesting a unique response to P4HB [70]. The co-expression of genes related to inflammatory, pathogen response, and the anti-inflammatory phenotypes may suggest a unique *in vitro* polarization state elicited by the P4HB mesh under these culture conditions.

In wound healing, the M1-like phenotype is important during the acute phase. The transition to anti-inflammatory phenotypes (M2a-, M2c-like) help to advance the wound healing response towards tissue restoration and ECM deposition [71]. Therefore, the comparison between P4HB and PP and how this could affect the transcriptome signaling in *in vitro*-derived M*ϕ*s is critical to understanding the *in vivo* response and potential benefits. M*ϕ*s cultured on P4HB showed a distinct gene expression profile when the polarizing cytokines were added. A particular transcriptome response was seen in stimulated M2a-like macrophages cultured on the materials, as seen in figures 5(B) and (C). The M*ϕ*s cultured on PP and stimulated with IL4 and IL13 were not able to fully match the M2a-like M*ϕ*s in UBP. PP promoted a gene expression profile closer to M0 or M2c-like phenotypes when compared to P4HB (based on the genes targeted in the panels). Even though an *in vivo* study of P4HB and PP in a murine model showed M2-like cells around both biomaterials, a larger M2-like population surrounding P4HB based scaffolds was reported [34]. Our results not only support this M2-like shift by P4HB, but the data suggest that the subpopulation transcriptome closest resembles the M2a-like gene profile.

It is important to note that the human wound healing response is complicated and involves many diverse cell types, cytokines, and intricate signaling cascades. Although the present study considered a wide range of representative gene expression markers for M*ϕ*s, the study is limited to one immune cell under laboratory conditions. Also, the specific cytokine concentrations used to stimulate M*ϕ* polarization *in vitro* and the short culture duration may not fully recapitulate the phenotype and time scale typically observed *in vivo*. Even with these limitations, identifying large-scale transcriptome changes in key cells such as M*ϕ*s can enhance our understanding of biomaterial-cell interactions and help guide future use and material design. This study allowed us to further understand previously published *in vivo animal* studies by targeting the *in vitro* human M*ϕ* gene expression. The targeted gene expression panel used provided a good representation of genes associated with inflammatory and anti-inflammatory responses. Additionally, while the M*ϕ*s are a crucial component in wound healing, crosstalk between cell types affects the host response and should be considered in future studies. The overall outcomes in this study aligned to the response observed in clinical and *in vivo* studies. Moreover, the present study offers a new look into the *in vitro* responses of human M*ϕ* and valuable insights into the potential clinical response.

## 5. Conclusions

This work elucidates the changes in the targeted transcriptomic response of human M*ϕ*s to absorbable P4HB and permanent PP meshes *in vitro*. M0s cultured on P4HB resulted in a phenotype with both pro-inflammatory and anti-inflammatory transcriptome signature. Further, the targeted transcriptome profile from human M*ϕ*s aligns with the pathogen response and increased M2a-like M*ϕ* population as reported in the literature for other models.

## Supplementary Material

Supplemental Doc 1

Supplemental Doc 2

## Figures and Tables

**Table 1.** A summary of the biomaterials used in the materials screening of this study [31–3,7].

Biomaterial	Material composition	Shape	Degradation ability
GalaFLEX^®^	Poly-4-hydroxybutyrate (P4HB)	Monofilament mesh	Absorbable
PROLENE^®^	Polypropylene (PP)	Monofilament mesh	Permanent
Alloderm^®^	Acellular dermal matrix	Sheet	Absorbable
Mersilene^®^	Polyethylene terephthalate polyester	Multifilament mesh	Permanent
SERI^®^	Silk fibroin	Multifilament mesh	Absorbable
TIGR^®^	Two copolymers based on glycolide, lactide, and trimethylene carbonate	Multifilament mesh	Absorbable
VICRYL^®^	Polyglactin	Multifilament mesh	Absorbable

## References

[1] Baylon K 2017 Past, present and future of surgical meshes: a review Membranes 7 47 28829367 10.3390/membranes7030047 PMC5618132

[2] Boersema GS, Grotenhuis N, Bayon Y, Lange JF and Bastiaansen-Jenniskens YM 2016 The effect of biomaterials used for tissue regeneration purposes on polarization of macrophages Biores Open Access 5 6–14 26862468 10.1089/biores.2015.0041 PMC4744891

[3] Heymann F 2019 Polypropylene mesh implantation for hernia repair causes myeloid cell-driven persistent inflammation JCI Insight 4 10.1172/jci.insight.123862 PMC6413778 30674727

[4] Wolf MT 2014 Polypropylene surgical mesh coated with extracellular matrix mitigates the host foreign body response J. Biomed. Mater. Res. A 102 234–46 23873846 10.1002/jbm.a.34671 PMC3808505

[5] Adams WP Jr., Baxter R, Glicksman C, Mast BA, Tantillo M and Van Natta B W 2018 The use of poly-4-hydroxybutyrate (P4HB) scaffold in the ptotic breast: a multicenter clinical study Aesth. Surg. J 38 502–18 10.1093/asj/sjy022 29401215

[6] Roth JS 2018 Prospective evaluation of poly-4-hydroxybutyrate mesh in CDC class I/high-risk ventral and incisional hernia repair: 18 month follow-up Surg. Endosc 32 1929–36 29063307 10.1007/s00464-017-5886-1

[7] Yu D 2019 Comparison of Phasix, polypropylene, and primary closure of the abdominal donor site after bilateral free flap breast reconstruction: long-term evaluation of abdominal hernia and bulge formation Microsurgery 40 434–9 31815314 10.1002/micr.30541

[8] Krzyszczyk P, Schloss R, Palmer A and Berthiaume F 2018 The role of macrophages in acute and chronic wound healing and interventions to promote pro-wound healing phenotypes Front. Physiol 9 419 29765329 10.3389/fphys.2018.00419 PMC5938667

[9] Butterfield TA, Best TM and Merrick MA 2006 The dual roles of neutrophils and macrophages in inflammation: a critical balance between tissue damage and repair J. Athl. Train 41 457–65 (https://pubmed.ncbi.nlm.nih.gov/17273473/) 17273473 PMC1748424

[10] Julier Z, Park AJ, Briquez PS and Martino MM 2017 Promoting tissue regeneration by modulating the immune system Acta Biomater 53 13–28 28119112 10.1016/j.actbio.2017.01.056

[11] Koh TJ and DiPietro LA 2011 Inflammation and wound healing: the role of the macrophage Expert Rev. Mol. Med 13 e23 21740602 10.1017/S1462399411001943 PMC3596046

[12] Lucas T, Waisman A, Ranjan R, Roes J, Krieg T, Müller W, Roers A and Eming SA 2010 Differential roles of macrophages in diverse phases of skin repair J. Immunol 184 3964–77 20176743 10.4049/jimmunol.0903356

[13] Gaffney L 2017 Macrophages’ role in tissue disease and regeneration Results Probl. Cell Differ 62 245–71 28455712 10.1007/978-3-319-54090-0_10

[14] Kim SY and Nair MG 2019 Macrophages in wound healing: activation and plasticity Immunol. Cell Biol 97 258–67 30746824 10.1111/imcb.12236 PMC6426672

[15] Miserez M, Jairam AP, Boersema GSA, Bayon Y, Jeekel J and Lange JF 2019 Resorbable synthetic meshes for abdominal wall defects in preclinical setting: a literature review J. Surg. Res 237 67–75 30710881 10.1016/j.jss.2018.11.054

[16] Orecchioni M, Ghosheh Y, Pramod AB and Ley K 2020 Corrigendum: macrophage polarization: different gene signatures in M1(LPS+) vs. classically and M2(LPS-) vs. alternatively activated macrophages Front. Immunol 11 234 32161587 10.3389/fimmu.2020.00234 PMC7053374

[17] Murray PJ 2014 Macrophage activation and polarization: nomenclature and experimental guidelines Immunity 41 14–20 25035950 10.1016/j.immuni.2014.06.008 PMC4123412

[18] Roszer T 2015 Understanding the mysterious M2 macrophage through activation markers and effector mechanisms Mediators Inflamm 2015 816460 26089604 10.1155/2015/816460 PMC4452191

[19] Mosser DM and Edwards JP 2008 Exploring the full spectrum of macrophage activation Nat. Rev. Immunol 8 958–69 19029990 10.1038/nri2448 PMC2724991

[20] Vogel DY, Glim JE, Stavenuiter AWD, Breur M, Heijnen P, Amor S, Dijkstra CD and Beelen RHJ 2014 Human macrophage polarization in vitro: maturation and activation methods compared Immunobiology 219 695–703 24916404 10.1016/j.imbio.2014.05.002

[21] Spiller KL 2016 Differential gene expression in human, murine, and cell line-derived macrophages upon polarization Exp. Cell Res 347 1–13 26500109 10.1016/j.yexcr.2015.10.017

[22] Wrona EA, Sun B, Romero-Torres S and Freytes DO 2019 Effects of polarized macrophages on the in vitro gene expression after co-culture of human pluripotent stem cell-derived cardiomyocytes J. Immunol. Regener. Med 4 100018

[23] Spiller KL, Anfang RR, Spiller KJ, Ng J, Nakazawa KR, Daulton JW and Vunjak-Novakovic G 2014 The role of macrophage phenotype in vascularization of tissue engineering scaffolds Biomaterials 35 4477–88 24589361 10.1016/j.biomaterials.2014.02.012 PMC4000280

[24] Martinez FO, Gordon S, Locati M and Mantovani A 2006 Transcriptional profiling of the human monocyte-to-macrophage differentiation and polarization: new molecules and patterns of gene expression J. Immunol 177 7303–11 17082649 10.4049/jimmunol.177.10.7303

[25] O’Neill JD, Freytes DO, Anandappa AJ, Oliver JA and Vunjak-Novakovic GV 2013 The regulation of growth and metabolism of kidney stem cells with regional specificity using extracellular matrix derived from kidney Biomaterials 34 9830–41 24074840 10.1016/j.biomaterials.2013.09.022 PMC3835733

[26] Wang H 2016 NanoStringDiff: a novel statistical method for differential expression analysis based on NanoString nCounter data Nucleic Acids Res 44 e151 27471031 10.1093/nar/gkw677 PMC5175344

[27] Vandesompele J, De Preter K, Pattyn F, Poppe B, Van Roy N, De Paepe A and Speleman F 2002 Accurate normalization of real-time quantitative RT-PCR data by geometric averaging of multiple internal control genes Genome Biol 3 RESEARCH0034 12184808 10.1186/gb-2002-3-7-research0034 PMC126239

[28] R Core Team 2019 R: A Language and Environment for Statistical Computing (Vienna: R Foundation for Statistical Computing)

[29] Gu Z, Eils R and Schlesner M 2016 Complex heatmaps reveal patterns and correlations in multidimensional genomic data Bioinformatics 32 2847–9 27207943 10.1093/bioinformatics/btw313

[30] Wickham H 2016 ggplot2 2nd edn R Use (Cham: Springer International Publishing)

[31] Cortes RA 2008 Biomaterials and the evolution of hernia repair I: the history of biomaterials and the permanent meshes Surgery: Basic Science and Clinical Evidence ed Norton JAet al (New York: Springer) pp 2291–304

[32] Jewell M, Daunch W, Bengtson B and Mortarino E 2015 The development of SERI(R) surgical scaffold, an engineered biological scaffold Ann. N. Y. Acad. Sci 1358 44–55 26376101 10.1111/nyas.12886

[33] Liu L 2016 The use of Vicryl mesh in a porcine model to assess its safety as an adjunct to posterior fascial closure during retromuscular mesh placement Hernia 20 289–95 26886013 10.1007/s10029-016-1469-7

[34] Pineda Molina C 2019 Comparison of the host macrophage response to synthetic and biologic surgical meshes used for ventral hernia repair J. Immunol. Regener. Med 3 13–25

[35] Todros S, Pavan PG and Natali AN 2017 Synthetic surgical meshes used in abdominal wall surgery: part I-materials and structural conformation J. Biomed. Mater. Res. B 105 689–99 10.1002/jbm.b.33586 26671827

[36] Williams SF, Martin DP and Moses AC 2016 The history of GalaFLEX P4HB scaffold Aesth. Surg. J 36 S33–42 10.1093/asj/sjw141 PMC5070449 27697885

[37] Xu H, Wan H, Sandor M, Qi S, Ervin F, Harper JR, Silverman RP and McQuillan DJ 2008 Host response to human acellular dermal matrix transplantation in a primate model of abdominal wall repair Tissue Eng. A 14 2009–19 10.1089/ten.tea.2007.0316 18593339

[38] Logan Ellis H, Asaolu O, Nebo V and Kasem A 2016 Biological and synthetic mesh use in breast reconstructive surgery: a literature review World J. Surg. Oncol 14 121 27102580 10.1186/s12957-016-0874-9 PMC4839154

[39] Cobb WS 2018 A current review of synthetic meshes in abdominal wall reconstruction Plast. Reconstr. Surg 142 64S–71S 30138270 10.1097/PRS.0000000000004857

[40] D’Angelo W, Dziki J and Badylak SF 2019 The challenge of stress incontinence and pelvic organ prolapse: revisiting biologic mesh materials Curr. Opin. Urol 29 437–42 31083010 10.1097/MOU.0000000000000645

[41] Lak KL and Goldblatt MI 2018 Mesh selection in abdominal wall reconstruction Plast. Reconstr. Surg 142 99S–106S 30138277 10.1097/PRS.0000000000004862

[42] Petro CC and Rosen MJ 2018 A current review of long-acting resorbable meshes in abdominal wall reconstruction Plast. Reconstr. Surg 142 84S–91S 30138274 10.1097/PRS.0000000000004859

[43] Anderson JM, Defife K, Mcnally A, Collier T and Jenney C 1999 Monocyte, macrophage and foreign body giant cell interactions with molecularly engineered surfaces J. Mater.Sci. Mater. Med 10 579–88 15347970 10.1023/a:1008976531592

[44] Chen S, Jones JA, Xu Y, Low H-Y, Anderson JM and Leong KW 2010 Characterization of topographical effects on macrophage behavior in a foreign body response model Biomaterials 31 3479–91 20138663 10.1016/j.biomaterials.2010.01.074 PMC2837101

[45] Rostam HM, Singh S, Salazar F, Magennis P, Hook A, Singh T, Vrana NE, Alexander MR and Ghaemmaghami AM 2016 The impact of surface chemistry modification on macrophage polarisation Immunobiology 221 1237–46 27349596 10.1016/j.imbio.2016.06.010

[46] Sridharan R, Cavanagh B, Cameron AR, Kelly DJ and O’Brien FJ 2019 Material stiffness influences the polarization state, function and migration mode of macrophages Acta Biomater 89 47–59 30826478 10.1016/j.actbio.2019.02.048

[47] Deeken CR, Abdo MS, Frisella MM and Matthews BD 2011 Physicomechanical evaluation of polypropylene, polyester, and polytetrafluoroethylene meshes for inguinal hernia repair J. Am. Coll. Surg 212 68–79 21115372 10.1016/j.jamcollsurg.2010.09.012

[48] Deeken CR and Lake SP 2017 Mechanical properties of the abdominal wall and biomaterials utilized for hernia repair J. Mech. Behav. Biomed. Mater 74 411–27 28692907 10.1016/j.jmbbm.2017.05.008

[49] Kwon KA, Shipley RJ, Edirisinghe M, Ezra DG, Rose GE, Rayment AW, Best SM and Cameron RE 2014 Microstructure and mechanical properties of synthetic brow-suspension materials Mater. Sci. Eng. C 35 220–30 10.1016/j.msec.2013.10.031 24411372

[50] Deeken CR and Matthews BD 2013 Characterization of the mechanical strength, resorption properties, and histologic characteristics of a fully absorbable material (poly-4-hydroxybutyrate-PHASIX mesh) in a porcine model of hernia repair ISRN Surg 2013 238067 23781348 10.1155/2013/238067 PMC3679684

[51] Williams SF, Rizk S and Martin DP 2013 Poly-4-hydroxybutyrate (P4HB): a new generation of resorbable medical devices for tissue repair and regeneration Biomed. Tech 58 439–52 10.1515/bmt-2013-0009 23979121

[52] Banyard DA, Bourgeois JM, Widgerow AD and Evans GRD 2015 Regenerative biomaterials: a review Plast. Reconstr. Surg 135 1740–8 26017603 10.1097/PRS.0000000000001272

[53] Eickhoff RM 2019 Improved biocompatibility of profiled sutures through lower macrophages adhesion J. Biomed. Mater. Res. B 107 1772–8 10.1002/jbm.b.34269 30452123

[54] Grotenhuis N, Bayon Y, Lange JF, Van Osch GJVM and Bastiaansen-Jenniskens YM 2013 A culture model to analyze the acute biomaterial-dependent reaction of human primary macrophages Biochem. Biophys. Res. Commun 433 115–20 23485466 10.1016/j.bbrc.2013.02.054

[55] Lock AM, Gao R, Naot D, Coleman B, Cornish J and Musson DS 2017 Induction of immune gene expression and inflammatory mediator release by commonly used surgical suture materials: an experimental in vitro study Patient Saf. Surg 11 16 28580016 10.1186/s13037-017-0132-2 PMC5452533

[56] Orenstein S, Qiao Y, Kaur M, Klueh U, Kreutzer D and Novitsky Y 2010 In vitro activation of human peripheral blood mononuclear cells induced by human biologic meshes J. Surg. Res 158 10–14 19853260 10.1016/j.jss.2009.05.033

[57] Panilaitis B 2003 Macrophage responses to silk Biomaterials 24 3079–85 12895580 10.1016/s0142-9612(03)00158-3

[58] Pineda Molina C 2019 4-hydroxybutyrate promotes endogenous antimicrobial peptide expression in macrophages Tissue Eng. A 25 693–706 10.1089/ten.TEA.2018.0377 30982430

[59] Utomo L, Boersema GSA, Bayon Y, Lange JF, van Osch GJVM and Bastiaansen-Jenniskens YM 2017 In vitro modulation of the behavior of adhering macrophages by medications is biomaterial-dependent Biomed. Mater 12 025006 28267684 10.1088/1748-605X/aa5cbc

[60] Scott JR, Deeken CR, Martindale RG and Rosen MJ 2016 Evaluation of a fully absorbable poly-4-hydroxy-butyrate/absorbable barrier composite mesh in a porcine model of ventral hernia repair Surg. Endosc. Other Interventional Tech 30 3691–701 10.1007/s00464-016-5057-9 PMC4992027 27369286

[61] Liao X 2011 Kruppel-like factor 4 regulates macrophage polarization J. Clin. Invest 121 2736–49 21670502 10.1172/JCI45444 PMC3223832

[62] Gensel JC, Kopper TJ, Zhang B, Orr MB and Bailey WM 2017 Predictive screening of M1 and M2 macrophages reveals the immunomodulatory effectiveness of post spinal cord injury azithromycin treatment Sci. Rep 7 40144 28057928 10.1038/srep40144 PMC5216345

[63] Hachim D, LoPresti ST, Yates CC and Brown BN 2017 Shifts in macrophage phenotype at the biomaterial interface via IL-4 eluting coatings are associated with improved implant integration Biomaterials 112 95–107 27760399 10.1016/j.biomaterials.2016.10.019 PMC5121003

[64] Lawrence T and Natoli G 2011 Transcriptional regulation of macrophage polarization: enabling diversity with identity Nat. Rev. Immunol 11 750–61 22025054 10.1038/nri3088

[65] Li H, Jiang T, Li M-Q, Zheng X-L and Zhao G-J 2018 Transcriptional regulation of macrophages polarization by microRNAs Front. Immunol 9 1175 29892301 10.3389/fimmu.2018.01175 PMC5985397

[66] Lake SP, Stoikes NFN, Badhwar A and Deeken CR 2019 Contamination of hybrid hernia meshes compared to bioresorbable Phasix Mesh in a rabbit subcutaneous implant inoculation model Ann. Med. Surg 46 12–6 10.1016/j.amsu.2019.08.004 PMC6710816 31467674

[67] Levy AS, Bogue J, Morrison KA, Lieberman MD, Pomp A and Spector JA 2016 Onlay mesh with P4HB provides superior outcomes in complex abdominal wall reconstruction J. Am. Coll. Surg 223 E108

[68] Messa CAT 2019 When the mesh goes away: an analysis of poly-4-hydroxybutyrate mesh for complex hernia repair. Plast Reconstr. Surg. Glob. Open 7 e2576 31942324 10.1097/GOX.0000000000002576 PMC6908335

[69] Pineda Molina C, Hussey GS, Liu A, Eriksson J, D’Angelo WA and Badylak SF 2020 Role of 4-hydroxybutyrate in increased resistance to surgical site infections associated with surgical meshes Biomaterials 267 120493 33202331 10.1016/j.biomaterials.2020.120493

[70] Tyner JW 2005 CCL5-CCR5 interaction provides antiapoptotic signals for macrophage survival during viral infection Nat. Med 11 1180–7 16208318 10.1038/nm1303 PMC6322907

[71] Witte MB and Barbul A 1997 General principles of wound healing Surg. Clin. North Am 77 509–28 9194878 10.1016/s0039-6109(05)70566-1

