# Supplementary material for: Transcriptome-targeted analysis of human peripheral blood-derived macrophages when cultured on biomaterial meshes
Source: Biomed Mater. Author manuscript; Available in PMC 2024 Dec 9. (PMC11626613; doi:10.1088/1748-605X/abdbdb)

Supporting Information

Transcriptome-Targeted Analysis of Human Peripheral Blood-Derived Macrophages When Cultured on Biomaterial Meshes

Camilo Mora-Navarro[1,2], Emily W. Ozpinar[1,2], Daphne Sze[1,2], David P. Martin[3] and Donald O. Freytes[1,2**]

[1]The Joint Department of Biomedical Engineering, North Carolina State University and University of North Carolina-Chapel Hill, Raleigh, NC

[2]The Comparative Medicine Institute, North Carolina State University, Raleigh, NC

[3]Tepha, Inc, Lexington, MA

**Corresponding Author:

Donald O. Freytes, Ph.D.
Joint Department of Biomedical Engineering
North Carolina State University
University of North Carolina-Chapel Hill
4208D Engineering Building III
Campus Box 7115
Raleigh, NC 27695
dofreyte@ncsu.edu
dfreytes@unc.edu
Office: 919-513-7933

Supplemental Figure S1-S6

Supplemental Figure S1. FACS for confirmation of CD14+ cells after isolation from peripheral blood mononuclear cells.

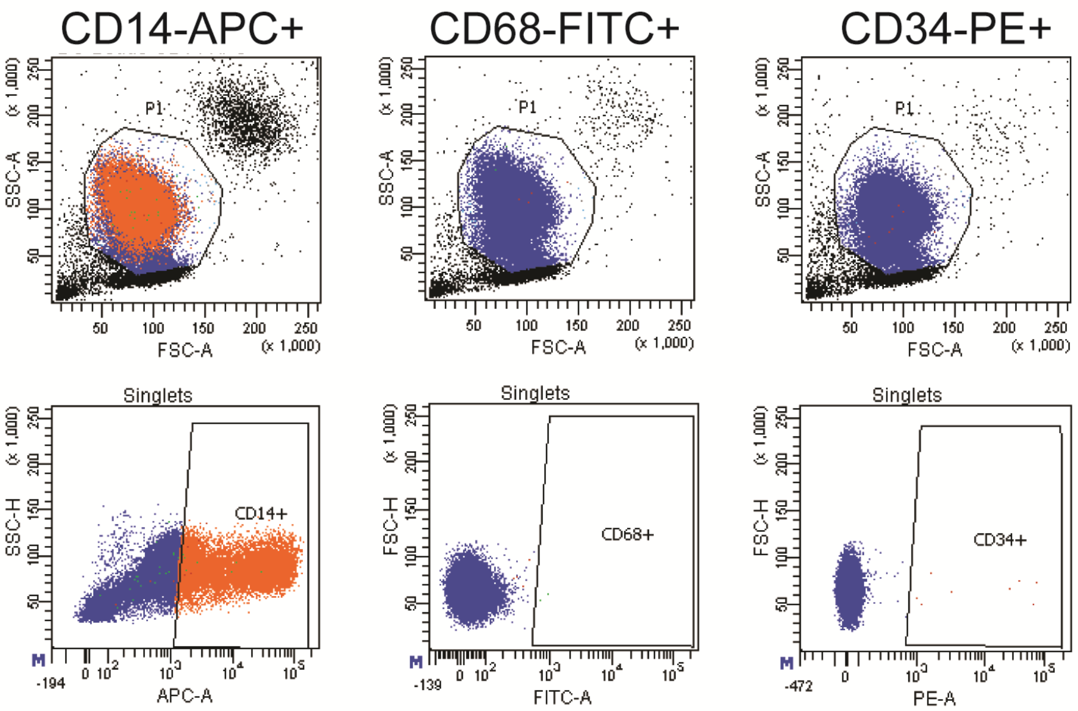

FACS provide evidence on the positive detection of CD14+ as feature marker for peripheral blood monocytes, which were further activated to macrophages (Mϕ).

Supplemental Figure S2. Top 10 uniquely differentially regulated genes regulated for M1, M2a and M2c-like phenotypes in macrophages (Mϕ) cultured on UBP across three donors. Colors represent the Log2 fold change value referenced to non-polarized M0 UBP control corresponding to the same donor and gene. The selection was based on the absolute values of the Log2 fold change with p-values < 0.05 for each polarization. M0 (Mϕ+M-CSF), M1 (LPS, IFNγ), M2a (IL4, IL13), and M2c (IL10).

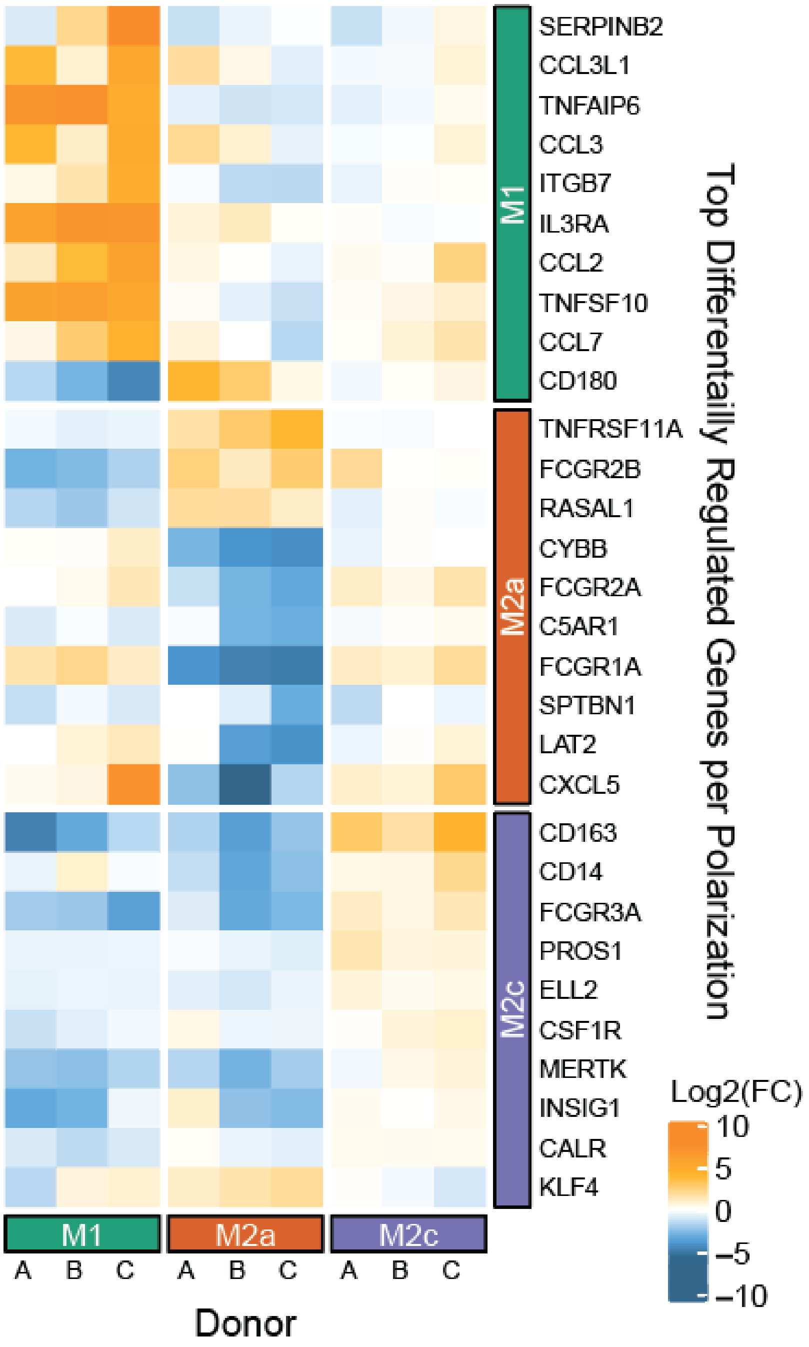

Supplemental Figure S3. PCA of M0s (Mϕ+M-CSF) cultured on various biomaterials. A) Dim 1 - Dim 2. B) Dim 1 - Dim 3. C) Dim 2 - Dim 3. D) PCA of Dim 1 - Dim 3 including Fibrin. Each point represents the mean coordinates of the individual samples for each biomaterial. Each sample is the biological average from one reading of three replicates pooled together.

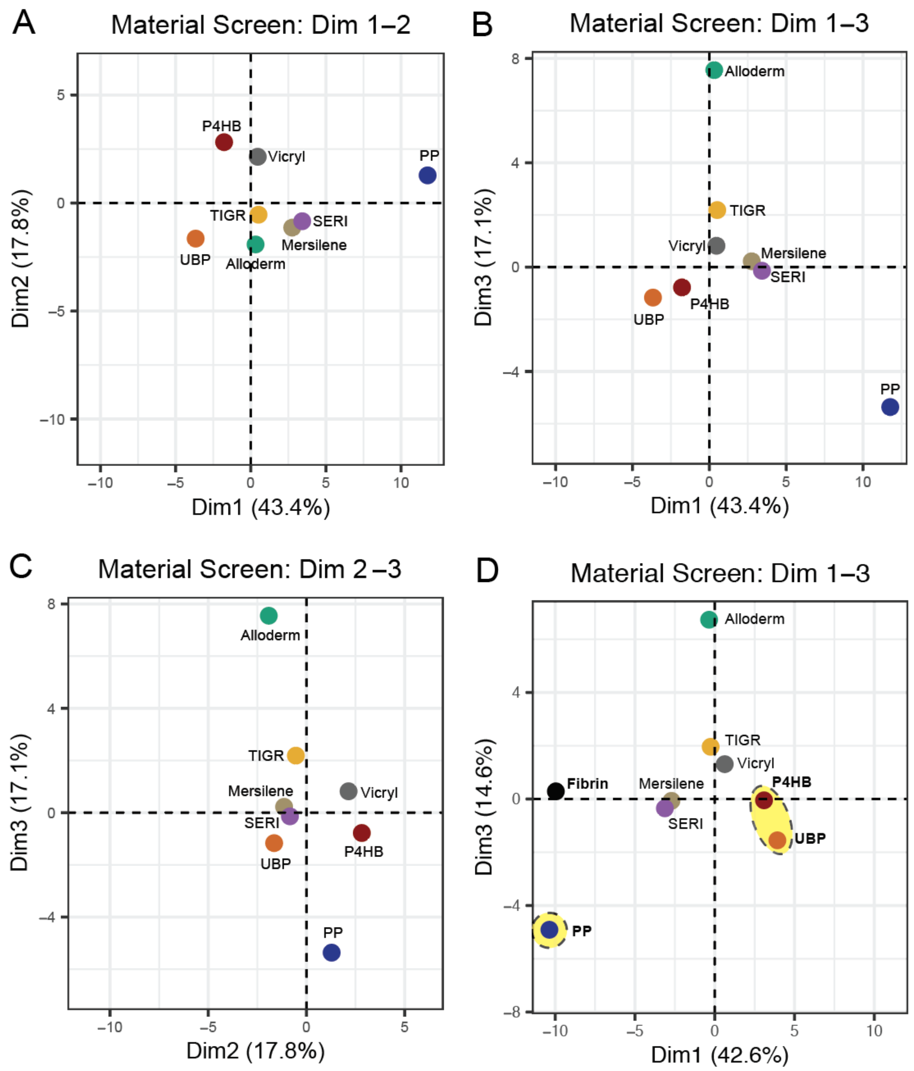

In Figure S3 A-C, we see dimension 1,2 and 3 for the PCA for the various biomaterials. When culturing M0s on these biomaterials, fibrin clots were used to attach the biomaterial to the culture plate. Figure S3 D shows dimensions 1-3 of the PCA for the biomaterials including a M0s in fibrin control to assess if using these fibrin clots affected the results. Fibrin localizes to the far left and does not group with the other biomaterials indicating that changes in the transcriptome cause by biomaterials are independent of fibrin. Furthermore, the inclusion of fibrin in the PCA does not drastically affect the relative clustering of the other biomaterials seen in Figure 1 C and S3 B.

Supplemental Figure S4. Volcanos plots referencing genes in ECM remodeling annotation for A) M0 P4HB ref. M0 UBP, B) M1 P4HB ref. M0 UBP, and C) M1 UBP ref. M0 UBP. The data represents n=3 independent biological replicates. “- - -” and “. . .” represent the alpha for -Log10 adj. p-values equal to 0.01 and 0.05 respectively.

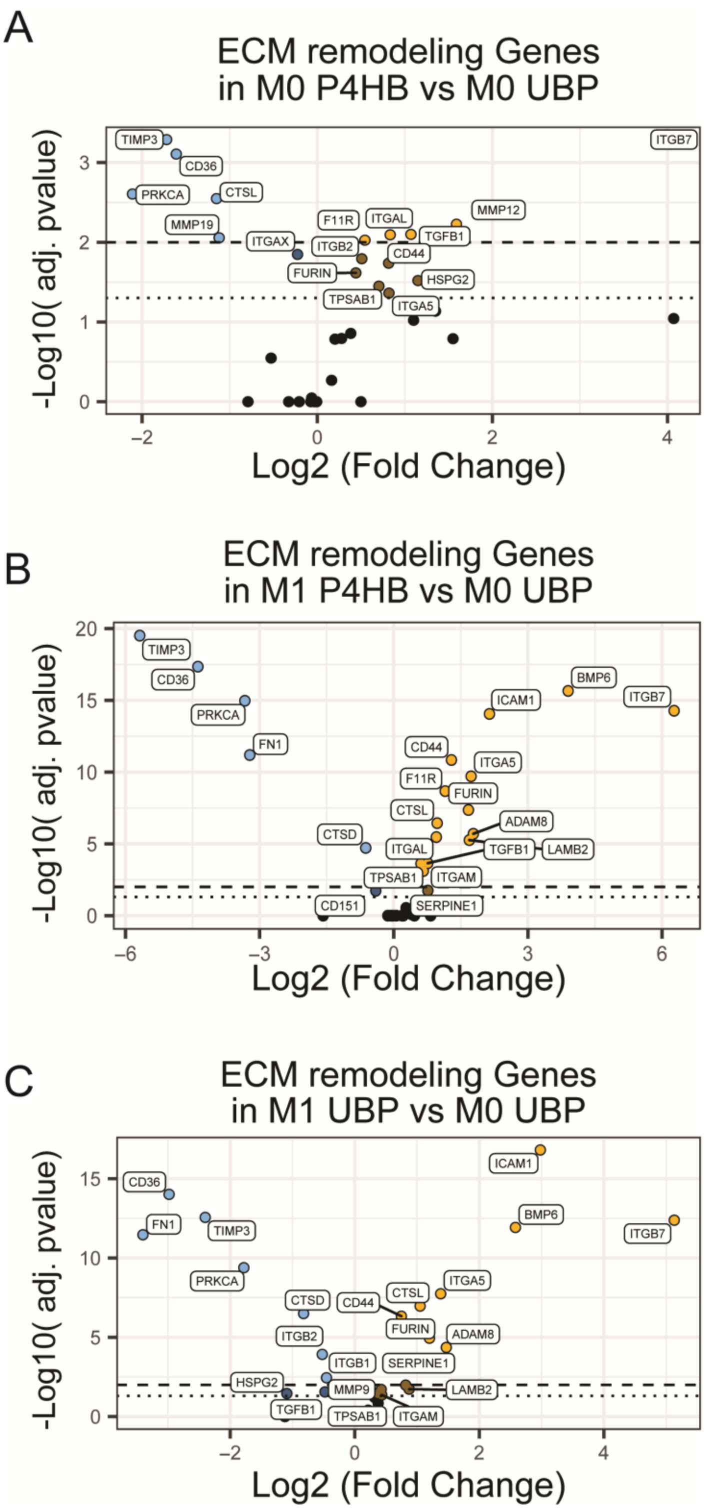

Supplemental Figure S5. Volcanos plot referencing genes in Cell migration and adhesion annotation for M0 P4HB ref. M0 UBP. The data represents n=3 independent biological replicates. “- - -” and “. . .” represent the alpha for -Log10 adj. p-values equal to 0.01 and 0.05 respectively.

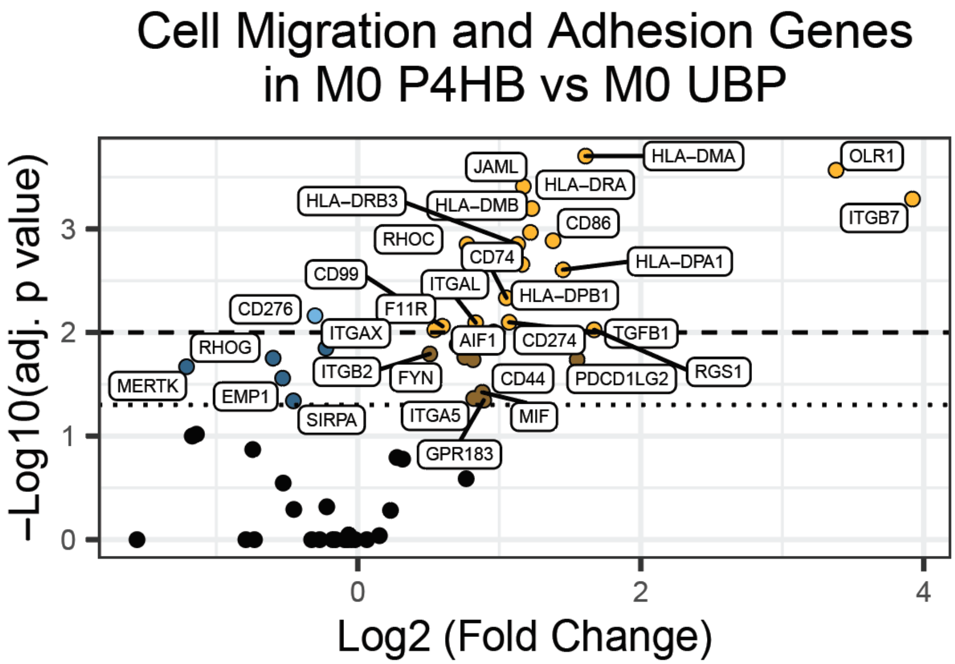

Supplemental Figure S6. Cell membrane staining of M0, M1, M2a and M2c-like phenotype Mϕs cultured on P4HB and PP. For each condition left images are transmitted light, images in the middle plasma membrane stained with CellMask™ Deep Red, the right images are the merged. Scale bar = 130 um. M0 (Mϕ+M-CSF), M1 (LPS, IFNγ), M2a (IL4, IL13), and M2c (IL10).

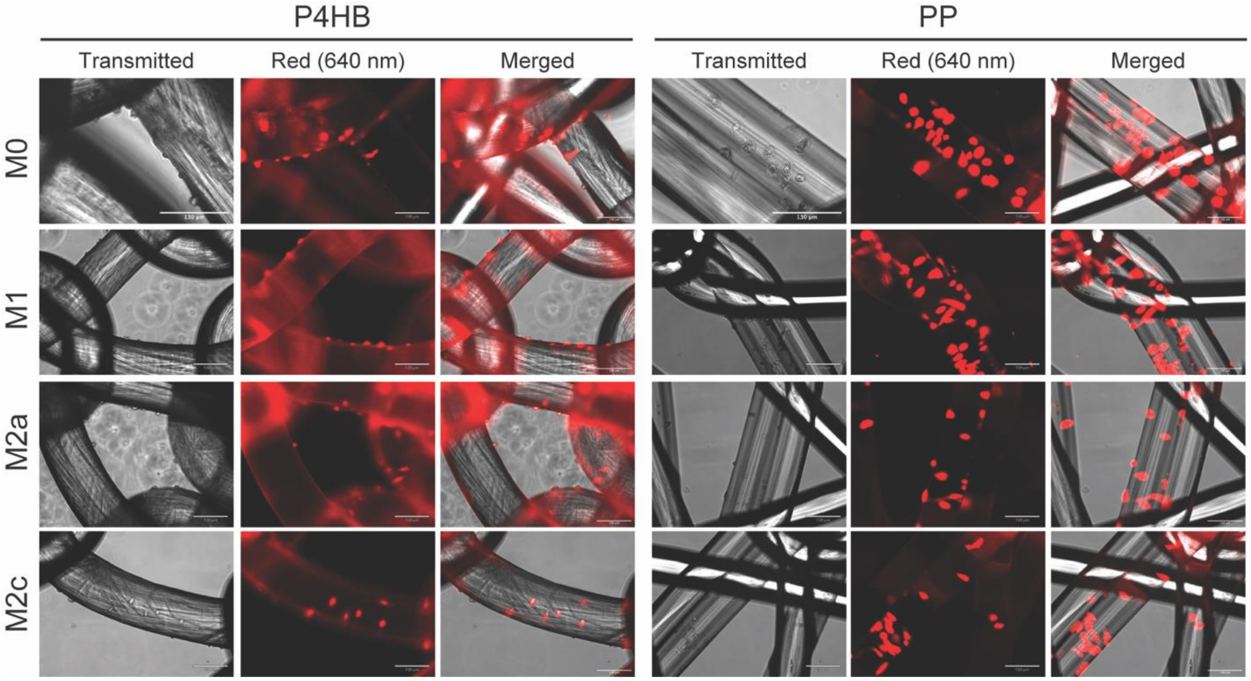

Supplement: Supplemental Doc 1 [file NIHMS2036563-supplement-Supplemental_Doc_1.pdf]
